# IGF-1 Upregulates Biglycan and Decorin by Increasing Translation and Reducing ADAMTS5 Expression

**DOI:** 10.3390/ijms22031403

**Published:** 2021-01-30

**Authors:** Hanon Lee, Jiyeong Lim, Jang-Hee Oh, Soyun Cho, Jin Ho Chung

**Affiliations:** 1 Department of Biomedical Sciences, Seoul National University Graduate School, Seoul 03080, Korea; iamhanon@gmail.com (H.L.); jidjiyeong@gmail.com (J.L.); 2 Department of Dermatology, Seoul National University College of Medicine, Seoul 03080, Korea; coolmox@gmail.com (J.-H.O.); sycho@snu.ac.kr (S.C.); 3 Institute of Human-Environment Interface Biology, Medical Research Center, Seoul National University, Seoul 03080, Korea; 4 Laboratory of Cutaneous Aging Research, Biomedical Research Institute, Seoul National University Hospital, Seoul 03080, Korea; 5 Department of Dermatology, SMG-SNU Boramae Medical Center, Seoul 07061, Korea

**Keywords:** insulin-like growth factor-1 (IGF-1), proteoglycan (PG), biglycan, decorin, a disintegrin and metalloproteinase with thrombospondin motifs 5 (ADAMTS5), skin aging

## Abstract

Proteoglycan (PG) is a glycosaminoglycan (GAG)-conjugated protein essential for maintaining tissue strength and elasticity. The most abundant skin PGs, biglycan and decorin, have been reported to decrease as skin ages. Insulin-like growth factor-1 (IGF-1) is important in various physiological functions such as cell survival, growth, and apoptosis. It is well known that the serum level of IGF-1 decreases with age. Therefore, we investigated whether and how IGF-1 affects biglycan and decorin. When primary cultured normal human dermal fibroblasts (NHDFs) were treated with IGF-1, protein levels of biglycan and decorin increased, despite no difference in mRNA expression. This increase was not inhibited by transcription blockade using actinomycin D, suggesting that it is mediated by IGF-1-induced enhanced translation. Additionally, both mRNA and protein expression of ADAMTS5, a PG-degrading enzyme, were decreased in IGF-1-treated NHDFs. Knockdown of ADAMTS5 via RNA interference increased protein expression of biglycan and decorin. Moreover, mRNA and protein expression of ADAMTS5 increased in aged human skin tissues compared to young tissue. Overall, IGF-1 increases biglycan and decorin, which is achieved by improving protein translation to increase synthesis and preventing ADAMTS5-mediated degradation. This suggests a new role of IGF-1 as a regulator for biglycan and decorin in skin aging process.

## 1. Introduction

Proteoglycans (PGs) consist of a core protein attached by one or more covalently linked glycosaminoglycan (GAG) chains. GAGs can be classified into six types including dermatan sulfate (DS), chondroitin sulfate, keratan sulfate, heparan sulfate, heparin, and hyaluronic acid with their specific disaccharide units [1]. With the exception hyaluronic acid, GAGs are attached to the core protein and enzymatically sulfated. Thus they are highly negatively-charged, consequently able to retain the water molecules.

Biglycan and decorin, members of small leucine-rich proteoglycans [2], are known as the most abundant PGs in the skin. They are primarily expressed in the dermis, although biglycan is somewhat observed in the epidermis. Decorin consists of a core protein (45 kDa) with one DS chain [3,4]. This intact form of decorin is discovered at approximately 70–100 kDa in human skin or in cultured normal human dermal fibroblasts (NHDFs), and proteolytic fragment forms are observed in the adult dermis [5]. Alternatively, biglycan is composed of a core protein (40 kDa) binding two DS chains. The intact form of biglycan with two DS chains and a mono-glycosylated form with one DS chain are detected approximately ~240 kDa and 70–100 kDa, respectively [6,7].

Skin aging is largely divided into intrinsic aging and photoaging. Intrinsic aging is characterized by a thinned epidermis and fine wrinkles, while photoaging shows deep wrinkles, skin laxity, telangiectasias, and lentigines depending on chronic sun exposure [8,9]. The main characteristic of the molecular mechanism is that the extracellular matrix, such as mature collagen, decreases. They are important for maintaining skin elasticity and tissue strength. Not only does their synthesis decrease, but also, degrading enzymes increase. A representative enzyme family is the matrix metalloproteinase (MMP) family, and MMP-1, an initial collagenase, exhibits a particularly increased expression in photoaged skin [10,11]. Biglycan and decorin are known to stabilize collagen fiber by regularly binding to collagen. In addition, decorin protects collagen from degradation by MMP-1. In decorin or biglycan knockout mouse studies, both of them exhibited thin dermis and fragile skin with irregular collagen fibril [8,12]. Moreover, mono-glycosylated and non-glycosylated forms (core protein) of biglycan or decorin have a weak ability to assist collagen fiber formation and maturation [13,14,15,16,17]. In the dermal matrix, elastic fibers are important structural components that contribute to skin elasticity. Decorin and biglycan interact with the components of elastic fiber, like tropoelastin, fibrillin-1, or microfibril-associated glycoproteins (MAGP) [18,19].

Moreover, biglycan and decorin interact with various signaling molecules, including transforming growth factor (TGF)-β, where decorin and biglycan bind to and sequester it, resulting in weakened TGF-β signaling [20,21]. Moreover, decorin is also known to bind to and downregulate signal transduction of platelet-derived growth factor (PDGF), epidermal growth factor receptor (EGFR), Met, IGF-1 and IGF receptor 1 [22,23,24,25]. Biglycan is known to bind and enhance signal transduction of BMP2, 4, Wnt3a, and EGFR [26,27,28,29]. Biglycan and decorin are also reported to activate toll-like receptor (TLR) 2 and 4 in macrophages [2,30,31].

Insulin-like growth factor 1 (IGF-1) is one of the important growth factors in mammals, which regulates various physiological functions including cell survival, growth, and metabolism of several tissues [32,33]. The human IGF-1 consists of 70 amino acids and has a similar molecular structure to insulin. However, despite the structural similarity between IGF-1 and insulin, the capacity of IGF-1 binding to an insulin receptor shows only a 1% low affinity [34]. IGF-1 receptor (IGF-1R), a protein tyrosine kinase receptor, also shares a high homology with the insulin receptor. IGF-1 is produced in almost every human tissue including the skin, and is especially highly expressed in liver [35]. In addition, it is well known that the IGF-1 level is abundant from birth to puberty, and gradually decreases with age [36]. Reduced IGF-1 secretion in the elderly is believed to cause several symptoms of aging [37]. In the skin, IGF-1 is synthesized and secreted from dermal fibroblasts, not expressed by epidermal keratinocytes. Its amount is reduced in aged skin as well. Moreover, IGF-1R expression did not indicate differences between young and old skin; however, activated forms of IGF-1R caused by IGF-1 are only shown in young skin [34].

In previous studies, we found that, through immunostaining, the expression of biglycan and decorin is decreased in intrinsically aged sun-protected skin, compared with young skin [7,38]. However, the mechanisms regulating the expression of biglycan and decorin in skin aging are not well understood. Therefore, we hypothesized that IGF-1 is responsible for reduced biglycan and decorin expression in aged skin. In this study, we found that recombinant human IGF-1 increases the protein amount of biglycan and decorin in NHDFs. This is due to the increased translation by IGF-1, not the transcriptional regulation, and is also due to the decreased proteolysis through reduced expression of ADAMTS5 by IGF-1.

## 2. Results

### 2.1. IGF-1 Increases Protein Levels of Biglycan and Decorin, but Not mRNA Expression in NHDFs

**Figure 1:**
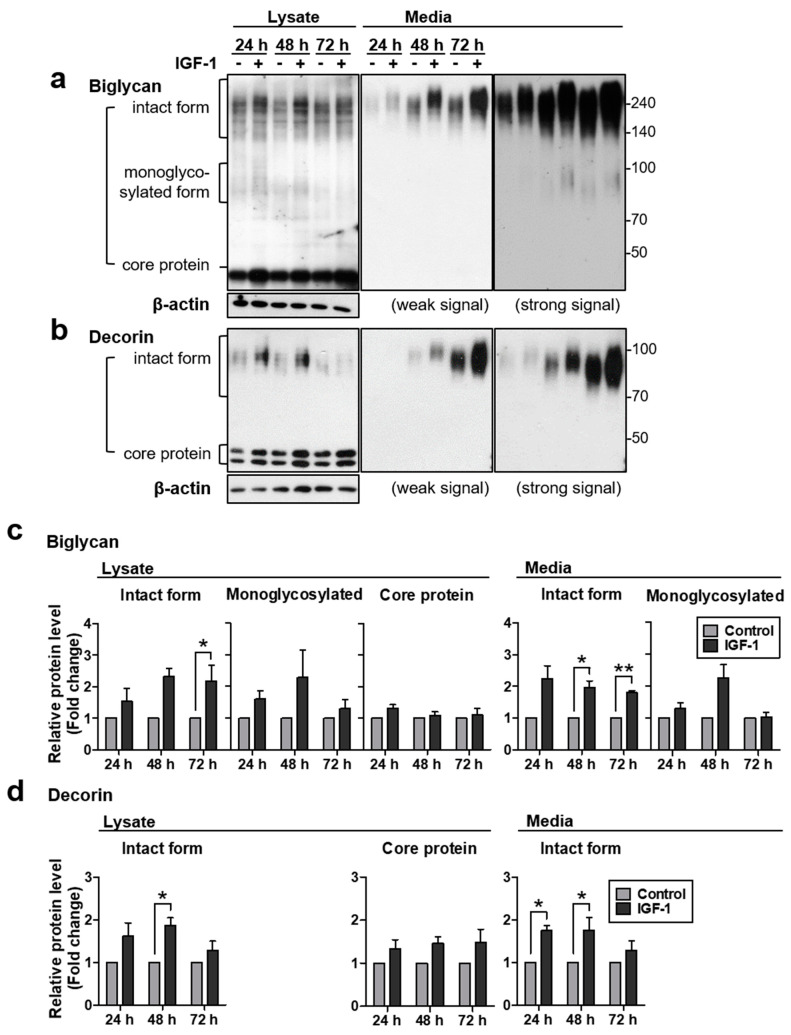
Insulin-like growth factor-1 (IGF-1) increased biglycan and decorin protein levels, but not mRNA expression in normal human dermal fibroblasts (NHDFs). Primary NHDFs were treated with 250 ng/mL of IGF-1 and harvested indicated time points. Protein levels of (**a**) biglycan and (**b**) decorin in cell lysates and conditioned media were analyzed by western blotting. (**c**,**d**) Relative protein amounts were analyzed using ImageJ. Band intensity was normalized to that of β-actin. Values represent the mean ± standard error of the mean (SEM) of data (*N* = 4, We performed 4 independent experiments using primary cells from 4 different individuals.). Statistical comparison was made using paired *t*-test. (* *p* < 0.05, ** *p* < 0.01).

The regulatory mechanism of biglycan and decorin expression in the skin is not well known. Therefore, in the present study, we investigated the effects of IGF-1 on biglycan and decorin expression in NHDFs. Treatment of NHDFs with 250 ng/mL of IGF-1 significantly increased biglycan and decorin protein levels in cell lysates and in conditioned medium at indicated time points (Figure 1). Biglycosylated biglycan and monoglycosylated decorin are the final forms of the intact proteoglycan, while the mono-glycosylated form biglycan or the core protein without GAG chains is a synthetic intermediate or degraded form. In our results, biglycan expression was observed in three forms in NHDFs: in cell lysate, the core protein form of biglycan (40 kDa) was most strongly detected, its intact biglycosylated form (>140 kDa) was also strongly detected, and its monoglycosylated form (>70 kDa) was faintly detected. In conditioned medium, the intact form was also strongly detected, and similarly, the monoglycosylated form was faintly detected, but no core protein was detected. All forms of biglycan protein levels were increased by IGF-1 treatment (Figure 1a) and the densitometry of its intact form showed that the biglycan protein level significantly increased at 72 h in cell lysate and at 48 h and 72 h in conditioned medium samples (Figure 1c). In a recent study that measured serum IGF-1 levels in over 2700 subjects, the median value in the 18-year-old group was 374.1 ng/mL and in the 70-year-old group was 92.7 ng/mL, reduced by 200–300 ng/mL [39]. Therefore, 250 ng/mL of IGF-1 seems to be in the acceptable range of physiological relevance.

The intact form of decorin (>70 kDa) also increased significantly in both cell lysate and conditioned medium, and its core protein form (doublet, <50 kDa) tended to increase in cell lysate, which was not detected in conditioned medium (Figure 1b). The densitometry of the decorin intact form showed that IGF-1-mediated increases were significant at 48 h in cell lysate and at 24 h and 48 h in conditioned medium samples (Figure 1d). In addition, the size of biglycan and decorin is increased. Proteoglycans, such as biglycan and decorin, vary in size depending on the length of GAG chain attached to the core protein. So their bands may have a wider range than general sharp shapes. Therefore, this size change is possibly due to the increase in the length of the GAG chain by IGF-1-mediated unknown regulatory mechanism.

Meanwhile, mRNA levels of biglycan and decorin did not change significantly at all time-points up to 72 h after rhIGF-1 (recombinant human IGF-1) treatment (Figure S1). These results suggested that the increase in the protein amount of biglycan and decorin by IGF-1 treatment may not be due to the induction of each mRNA expression, but due to post-transcriptional regulation.

### 2.2. IGF-1 Increases Protein Levels of Biglycan and Decorin by Upregulating Protein Translation in NHDFs

**Figure 2:**
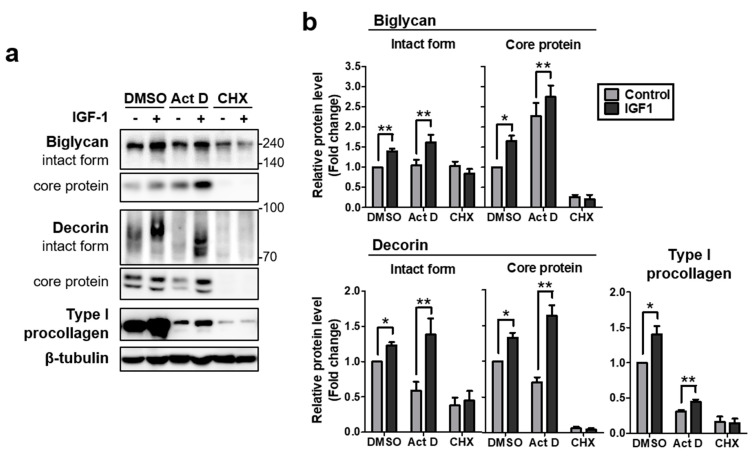
IGF-1 increased the protein levels of biglycan and decorin by upregulating protein translation in NHDFs. (**a**) Primary NHDFs were treated with actinomycin D (Act D, 1 µg/mL) or cycloheximide (CHX, 25 µg/mL) under basal conditions or in the presence of IGF-1 (250 ng/mL) for 18 h. Proteins of conditioned media and cell lysates were analyzed by western blotting. (**b**) Relative protein amounts were analyzed using ImageJ. Band intensity was normalized to that of β-tubulin. Values represent the mean ± SEM of data (*N* = 4). Statistical comparison was made using paired *t*-test. (* *p* < 0.05, ** *p* < 0.01).

As no mRNA induction of biglycan and decorin was observed, and IGF-1 is well known to increase protein translation [40], we examined whether IGF-1-mediated increases of biglycan and decorin protein levels were mediated by enhanced protein translation. Thus, we investigated the effects of actinomycin D (Act D) and cycloheximide (CHX) treatment, which inhibit transcription and translation, respectively. Cell lysates were harvested 18 h after Act D, CHX, or DMSO treatment in the absence (control) or presence of rhIGF-1. Thereafter, samples were analyzed by western blotting. In Act D-treated samples, the amounts of biglycan and decorin intact forms and core proteins were significantly increased by rhIGF-1 stimulation as in the DMSO control, whereas upregulation was impaired by CHX treatment. Type I procollagen also showed a similar pattern (Figure 2a,b), as previously reported [40]. These results suggested that the quantitative increase of biglycan and decorin proteins by IGF-1 treatment may be at least partially due to an IGF-1-induced increase in protein translation, rather than transcription.

### 2.3. IGF-1 Downregulates the Expression of ADAMTS5, and Knockdown of ADAMTS5 Resulted in Augmented Biglycan and Decorin Protein Levels in NHDFs

**Figure 3:**
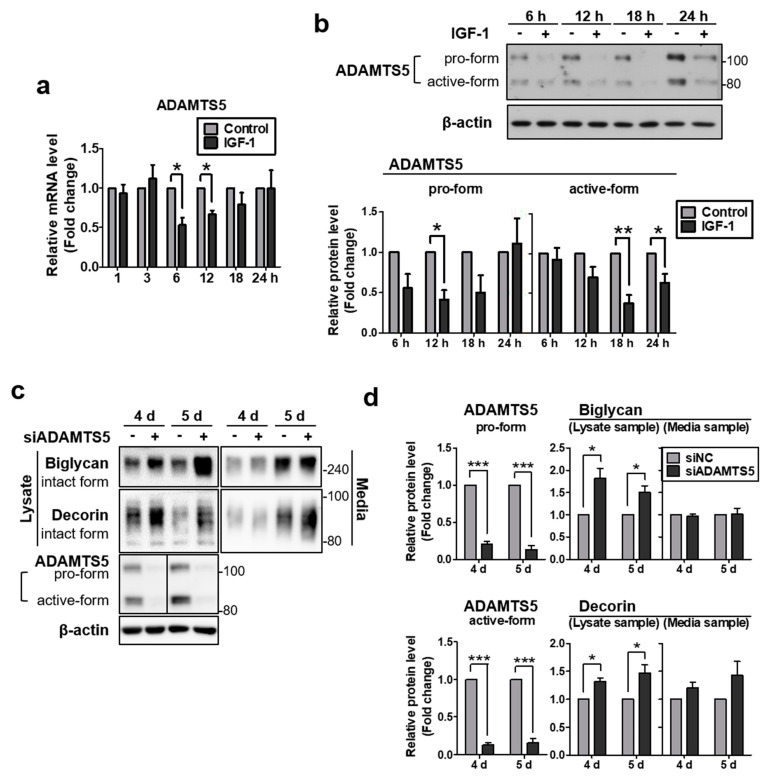
IGF-1 downregulated the expression of ADAMTS5, and knockdown of ADAMTS5 resulted in augmented biglycan and decorin protein levels in NHDFs. Total RNA was extracted and the expression of (**a**) ADAMTS5 mRNA was subsequently measured by quantitative real-time PCR and normalized to the expression of 36B4. (**b**) Protein levels of ADAMTS5 were measured by western blotting. (**c**) Primary NHDFs were treated with ADAMTS5 siRNA and harvested at indicated times after treatment. Proteins of conditioned media and cell lysates were analyzed by western blotting. (**d**) Relative protein amounts were analyzed using ImageJ. Band intensity was normalized to that of β-actin. Values represent the mean ± SEM of data (*N* = 4). Statistical comparison was made using paired *t*-test. (* *p* < 0.05, ** *p* < 0.01, *** *p* < 0.001).

In addition to directly increasing the protein synthesis of biglycan and decorin, we examined whether IGF-1 is also involved in their breakdown mechanism. ADAMTS5 is the member of a disintegrin and metalloproteinase with thrombospondin motifs (ADAMTS) family and also known as aggrecanase2. Aside from its major ability to degrade aggrecan, one of the PGs, it has been reported for proteolytic activity against other PGs such as decorin, biglycan, and brevican [41]. Therefore, we investigated the change of ADAMTS5 expression by IGF-1 stimulation. Following rhIGF-1 treatment, mRNA levels of ADAMTS5 were significantly reduced at 6 and 8 h (Figure 3a). In protein analysis, the size of ADAMTS5 may vary for each cell type due to glycosylation, unprocessed pro-form (100–120 kDa) appears at the approximate predicted molecular mass (100 kDa) and the cleaved active-form has a smaller size (70–85 kDa) [42,43,44]. In our samples, two major forms of ADAMTS5 were observed and each band appeared to be pro-form and active-form. The protein levels were significantly decreased at 12 h for the pro-form and at 18 and 24 h for the active-form (Figure 3b). These results showed that the IGF-1-mediated reduction of ADAMTS5 expression is likely to participate in the upregulation of biglycan and decorin protein levels by IGF-1 treatment.

To investigate whether the increase of biglycan and decorin expression is associated with IGF-1-mediated downregulation of ADAMTS5 expression in NHDFs, protein level changes of biglycan and decorin were examined after ADAMTS5 knockdown using siRNA. Following ADAMTS5 siRNA transfection, conditioned media and cell lysates were harvested at 4 and 5 d. Protein downregulation of ADAMTS5 by siRNA transfection was confirmed in cell lysate samples by western blotting, compared to the negative control siRNA-transfected sample (Figure 3c,d). Moreover, the protein levels of biglycan and decorin showed a significant increase both at 4 and 5 d in cell lysate samples. However, those of conditioned media did not show significant differences. Our results indicated that the IGF-1-induced downregulation of ADAMTS5 expression may contribute to the IGF-1-induced increase of biglycan and decorin expression.

### 2.4. ADAMTS5 Expression Is Increased in the Dermis of Elderly People

**Figure 4:**
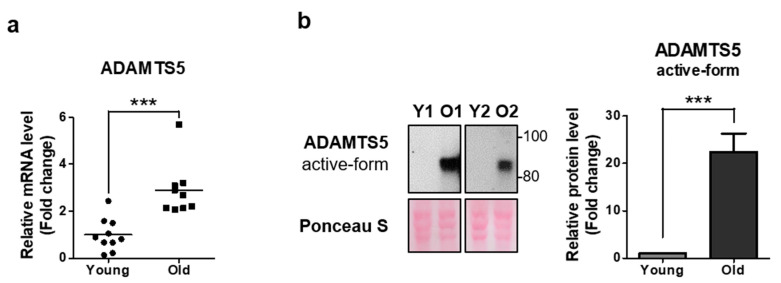
ADAMTS5 expression was increased in the dermis of elderly people. Total RNA was extracted in the dermis of young and aged sun-protected skin. Thereafter, the expression of (**a**) ADAMTS5 were measured by quantitative real-time PCR and normalized to the expression of 36B4 (*N* = 19). (**b**) ADAMTS5 protein expression was analyzed by western blotting. We compared the sun-protected buttock dermis in young and old people. (Y; Young, O; Old) Ponceau S panel represents the loading control. Band intensity was analyzed using ImageJ and normalized to that of Ponceau S. Values represent the mean ± SEM of relative fold change (*N* = 16). Statistical analyses were performed using Mann-Whitney *U* test. (*** *p* < 0.001).

In a previous study, we found that through immunostaining, biglycan expression decreased in aged sun-protected skin, compared with young skin [7]. Moreover, it has been reported that in the elderly, serum IGF-1 or IGF-1 secreted from dermal fibroblasts decreases [34,36]. Therefore, we could hypothesize that the reduced IGF-1 level in elderly subjects may result in an increase of ADAMTS5 protein level in aged skin. To confirm this, we investigated the expression of ADAMTS5 mRNA and protein in young and aged sun-protected skin. We observed that ADAMTS5 expression significantly increased in both mRNA (Figure 4a) and protein (Figure 4b) in aged skin. Therefore, it can be inferred that the reduction of serum IGF-1 levels with aging may be a possible cause for the increase of ADAMTS5 levels in aged dermis tissues, resulting in degradation of biglycan and decorin, leading to reduction in the amount of biglycan and decorin in aged dermal tissue.

## 3. Discussion

In this study, we investigated the regulatory effects of IGF-1 on the expression of biglycan and decorin. The importance of biglycan and decorin in the skin aging process has recently emerged. However, their regulatory mechanism has not been fully elucidated. Meanwhile, the serum IGF-1 level is known to decrease with age [34,45] and seems to be associated with skin wrinkling and facial aging [46]. In this regard, we have demonstrated the possibility of IGF-1 as a regulator in the skin aging process by regulating biglycan and decorin.

Firstly, we showed that IGF-1 upregulates the expression of biglycan and decorin by enhancing translation in NHDFs. It is well known that IGF-1 enhances translation via the PI3K-AKT-mTOR pathway [47,48,49]. However, reports of IGF-1-mediated regulation of biglycan and decorin have been focused on transcriptional change only, and the regulatory aspect seems to vary depending on cell type. In periodontal ligament fibroblasts, biglycan and decorin mRNA increased with IGF-1 treatment, but in gingival fibroblasts, only decorin mRNA increased while biglycan mRNA did not change [50]. Conversely, in MG63 osteosarcoma cells or the osteoblastic cell line, biglycan mRNA expression increased with IGF-1 treatment [51]. In chondrocytes, there were no mRNA expression changes for both biglycan and decorin [52]. In this study, protein expression of biglycan and decorin is upregulated by IGF-1 without transcriptional change in NHDFs. Thereafter, it was once again verified that an increase of their protein levels still occurred after blocking transcription by treatment with actinomycin D, whereas it was inhibited by treatment with a translation inhibitor, cycloheximide. These results suggest that IGF-1-mediated translation enhancement may contribute to the increase of biglycan and decorin protein levels. Furthermore, it has been known that the level of IGF-1 in serum is reduced by 200–300 (ng/mL) in elderly subjects [34,39,45]. Considering this together with our results, in which IGF-1 increases translation of biglycan and decorin, it can be deduced that the reduced expression of biglycan and decorin in intrinsically aged skin [7] may be partially due to the downregulated protein translation caused by a decrease in IGF-1 levels.

Secondly, this study suggests another regulatory mechanism of biglycan and decorin via the downregulation of ADAMTS5 by IGF-1 in addition to the strengthening of translation. The role of ADAMTS5 in aging-related diseases such as osteoarthritis is well known. This is because ADAMTS5 is a distinguished aggrecan-degrading protease actively studied in chondrocytes. However, as aggrecan is not a major PG in dermal fibroblasts, the role of ADAMTS5 in skin or dermal fibroblasts have been rarely reported. However, ADAMTS5 is also known to degrade other PGs including versican, biglycan, and decorin [53], which are the major PGs in human skin [4]. This implies the necessity for research on ADAMTS5 in human skin aging, and, in doing so, this study demonstrated that the increase of biglycan and decorin protein levels occurred in NHDFs according to the decrease of ADAMTS5 expression, which were mediated by IGF-1 treatment or by ADAMTS5 siRNA transfection. In chondrocytes, several studies have reported that the expression of ADAMTS5 reduced by IGF-1 treatment as an aggrecan-degrading enzyme, which is identical to our results [54,55]. Therefore, our results indicated that ADAMTS5 may participate in the degradation of biglycan and decorin in the skin, and reduced ADAMTS5 expression by IGF-1 may play roles in reducing degradation of biglycan and decorin.

Meanwhile, matrix-degrading enzymes, including collagenase and elastase (such as MMP-1 and 12), have long been known to play an important role in the skin aging process [56,57]. In recent studies, neutrophil elastases or granzyme B have been reported to break down decorin or both biglycan and decorin [11,58,59]. As biglycan and decorin play a role in the integrity of collagen fibers [8,12], their degradation may lead to collagen fiber decomposition, as well as skin aging. Some reports have demonstrated that the degradation of decorin promotes the decomposition of collagen fibers in the photo-aging process [11]. Thus, it is considered that degrading enzymes of biglycan and decorin can promote skin aging [60], and this study newly suggests possible roles of ADAMTS5 as a degrading enzyme of biglycan and decorin. This suggestion is supported by the fact that the expression of biglycan and decorin was increased by ADAMTS5 siRNA treatment.

Increase of biglycan and decorin by ADAMTS5 siRNA treatment was observed as significant in the cell lysate samples, but not in the conditioned media. In our opinion, it may be because the media is not the final destination of biglcan and decorin. They are finally incorporated into ECM by binding with other matrix proteins, like collagens, elastin, or fibrillins [31], resulting in insoluble matrix substances. In addition, when we extracted the cell lysates, we used a high concentration SDS extraction buffer with scraping. Because it contains a strong detergent, it may extract cell lysates and even at least some of insoluble matrix proteins. Therefore, the increased biglycan and decorin proteins in the lysate samples may be partly from the ECM, because ECM is their final destination to be accumulated. From this point of view, the reason why their increases were not clearly observed in the conditioned media may be because the media is not the destination of PG, but an intermediate stage of ECM. In addition, the effect of ADAMTS5 knock-down may be smaller than the case of IGF-1 treatment, because ADAMTS5 knock-down only reduces the protein degradation, and may not affect the PG production.

In addition to the in vitro study, both mRNA and protein expression of ADAMTS5 significantly increased in aged human buttock skin tissue, in particular, increase of the ADAMTS5 active-form indicates that degradation of various ADAMTS5 substrates like biglycan and decorin may actually be occurring in the dermis of the elderly. The cause of skin aging is not simple, and other unknown factors may work in combination. We presented one possibility that ADAMTS5 could be one of the new anti-skin aging targets, and it is suggested that inhibiting this enzyme may contribute to suppressing skin aging.

However, it should be considered that there are some limitations in our in vitro cellular system using adolescent foreskin-derived fibroblasts. Although experiments based on adolescent foreskin-derived fibroblasts are widely used, blind spots exist in that the fibroblasts can have body site-specific or age-dependent characteristics [61,62]. Using cells of a limited age range may have limitation that it cannot reflect the possibility that the response to IGF-1 may vary with age. Therefore, further investigation may be needed to apply it to the aging phenomenon.

Overall, IGF-1 treatment increases biglycan and decorin protein expression in cultured NHDFs. This is because biglycan and decorin synthesis increases directly by strengthening translation, and indirectly, ADAMTS5 is downregulated, resulting in less degradation of biglycan and decorin. This suggests that the reduction of IGF-1 in aging may contribute to the skin aging phenotype via downregulation of the protein levels of biglycan and decorin.

## 4. Materials and Methods

### 4.1. Study/Ethical Approval

Skin tissues were obtained and used for cell culture or tissue analysis, which is described in detail below. All procedures involving human subjects received prior approval from the Seoul National University Institutional Review Board, and all human subjects provided written informed consent. This study was conducted according to the principles described in the Declaration of Helsinki.

### 4.2. Cell Culture

Primary NHDFs were isolated from foreskin specimens of healthy male donors aged 10 to 19 years. Then, cells were cultured in Dulbecco’s modified Eagle’s media (DMEM, Welgene, Daegu, Korea), supplemented with 10% fetal bovine serum (FBS, Gibco, Rockville, MD, USA) and 1% penicillin-streptomycin (Gibco) in a humidified 5% CO2 atmosphere at 37 °C.

### 4.3. Treatment with Recombinant Human IGF-1 (rhIGF-1)

For treatment with rhIGF-1, NHDFs were starved in serum-free DMEM for 48 h. Thereafter, cells were treated with fresh serum-free DMEM containing 250 ng/mL of rhIGF-1 (R&D Systems Inc., Minneapolis, MN, USA) at indicated times, and harvested for mRNA and protein analysis.

### 4.4. Gene Silencing with siRNA

NHDFs were seeded and transfected simultaneously with non-targeted negative control siRNA (AccuTarget™ Negative control siRNA, Bioneer, Daejeon, Korea) or ADAMTS5 siRNA (Cat# 1002470, Bioneer) (300 nM) using G-fectin (Genolution, Seoul, Korea), according to the manufacturer’s instructions. At 24 h after transfection, media were replaced with serum-free DMEM. Cell lysates and cultured media were harvested for western blot analysis at indicated times after transfection.

### 4.5. Quantitative Real-Time PCR

Total RNA was isolated from cultured NHDFs using RNAiso Plus (Takara Bio Inc., Shiga, Japan). One microgram of total RNA was converted to cDNA using the First Strand cDNA Synthesis Kit (Thermo Fisher Scientific, Waltham, MA, USA). Quantitative real-time PCR was performed on a 7500 Real-Time PCR system (Applied Biosystems, Foster City, CA, USA) using TB green™ Premix Ex Taq™ (Cat# RR420A, Takara Bio) according to the manufacturer’s instructions. The primer sequences were listed in Table S1. The PCR conditions were 50 °C for 2 min, following by 40 cycles at 95 °C for 15 s, and 60 °C for 1 min. The data were analyzed by the 2^(−∆∆Ct)^ method and represented as fold changes of gene expression relative to 36B4.

### 4.6. Western Blot Analysis

To extract the protein for western blot analysis, cells were harvested by scraping on ice with 1× SDS-sample buffer containing protease and phosphatase inhibitor cocktails (Sigma-Aldrich, St. Louis, MO, USA) and then heated at 95 °C for 5 min. In addition, conditioned media were collected to detect secreted proteins. An equivalent volume of conditioned media from an equal number of cells were mixed with 4× SDS-sample buffer, and heated at 95 °C for 5 min. Equal amounts of samples were loaded and separated by SDS-PAGE and transferred onto nitrocellulose membranes. Following blocking with 5% skim milk, membranes were immunoblotted with primary anti-human decorin, biglycan (R&D Systems Inc., Minneapolis, MN, USA), β-actin (Thermo Fisher Scientific), β-tubulin (Santa Cruz Biotechnology, Santa Cruz, CA, USA), ADAMTS5 (GeneTex, Irvine, CA, USA), or monoclonal anti-procollagen type I amino-terminal extension peptide (SP1.D8) (Developmental Studies Hybridoma Bank, Iowa City, IA, USA) antibody, and polyclonal (GeneTex) or monoclonal secondary antibody (Santa Cruz Biotechnology), then detected using enhanced chemiluminescence (Thermo Fisher Scientific).

### 4.7. Human Skin Samples

To investigate the changes of ADAMTS5 mRNA or protein expression in human skin in vivo, we recruited 22 Korean volunteers and divided into the young and the elderly group. We analyzed 10 samples of young group (mean ± standard error of the mean (SEM) age, 28.2 ± 2.24 years; median, 26.5; range, 20–40 years) and 9 samples of elderly group (mean ± SEM age, 79.56 ± 2.40 years; median, 78; range, 70–94 years) for RNA analysis. For western blotting, 8 samples each of young group (mean ± SEM age, 27.5 ± 2.69 years; median, 23; range, 21–40 years) and of elderly group (mean ± SEM age, 81.38 ± 2.35 years; median, 79.5; range, 75–94 years) were analyzed. Biopsies were performed in the photo-protected buttock area and tissues were stored in liquid nitrogen immediately thereafter. Whole skin specimens were subsequently incubated in 55 °C phosphate-buffered saline (PBS) for 2 min, and separated into epidermis and dermis. Thereafter, the dermis was crushed to analyze mRNA and protein. In protein analysis, Ponceau S (Elpis-Biotech, Daejeon, Korea) was used to confirm loading control.

### 4.8. Statistical Analysis

Statistical analyses were performed using the paired t-test or Mann-Whitney U test. Results were represented as the mean ± SEM. *p*-values less than 0.05 were considered statistically significant.

## Data Availability

No publicly archived datasets were generated or analyzed during the current study.

## Supplementary Materials

The following are available online at https://www.mdpi.com/1422-0067/22/3/1403/s1, Figure S1: IGF-1 did not induce biglycan and decorin mRNA expression in NHDFs, Table S1: Primer sequences of human genes used for quantitative real-time PCR.

## Abbreviations

IGF-1	Insulin-like growth factor-1
ADAMTS5	a disintegrin and metalloproteinase with thrombospondin motifs 5
PG	proteoglycan
GAG	glycosaminoglycan
NHDFs	normal human dermal fibroblasts
DS	dermatan sulfate
MMP	matrix metalloproteinase
Act D	actinomycin D
CHX	cycloheximide
rhIGF-1	recombinant human IGF-1
siRNA	small interference RNA
