# Supplementary material for: IGF-1 Upregulates Biglycan and Decorin by Increasing Translation and Reducing ADAMTS5 Expression
Source: Int J Mol Sci. 2021 Jan 30;22(3):1403. doi: 10.3390/ijms22031403 (PMC7866853; doi:10.3390/ijms22031403)
Supplement: Supplementary file 1 [file ijms-22-01403-s001.pdf]

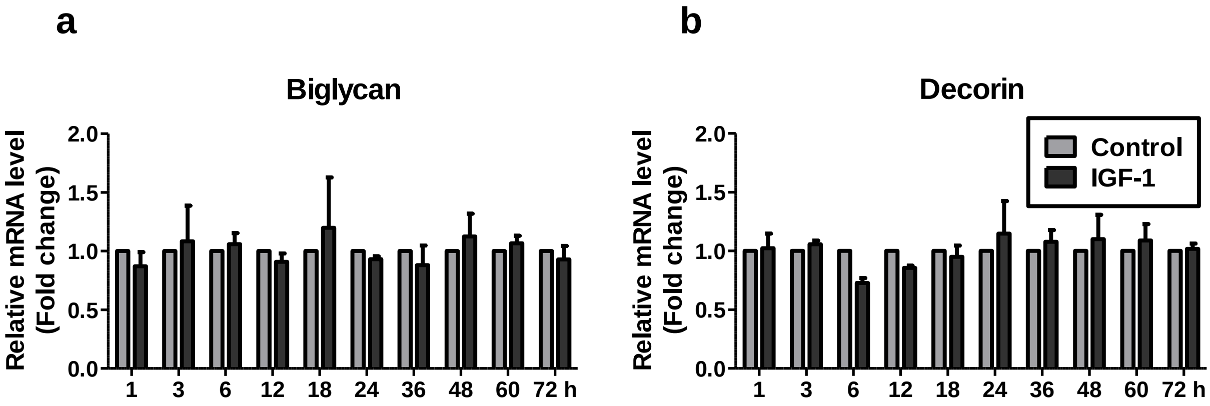

Figure S1. IGF-1 did not induce biglycan and decorin mRNA expression in NHDFs.

Primary NHDFs were treated with 250 ng/mL of IGF-1 and harvested according to the time course after treatment. Total RNA was extracted and the mRNA expression of biglycan (a) and decorin (b) in IGF-1-stimulated dermal fibroblasts was measured by quantitative real-time PCR, normalized with 36B4. Values represent the mean ± SEM of data (N = 4). Statistical comparison was made using paired *t*-test.

Table S1. Primer sequences of human genes used for quantitative real-time PCR

Target of primer	Sequence
**Biglycan**	Forward: 5’-GTC TAT CTG CAC TCC AAC AA-3’
	Reverse: 5’-TGG ATG GCC AGG CGG TCA GT-3’
**Decorin**	Forward: 5’-AGC TGA AGG AAT TGC CAG AA-3’
	Reverse: 5’-GCC ATT GTC AAC AGC AGA GA-3’
**36B4**	Forward: 5’-TCG ACA ATG GCA GCA TCT AC-3’
	Reverse: 5’-TGA TGC AAC AGT TGG GTA GC-3’
**ADAMTS5**	Forward: 5’-GCT ACT GCA CAG GGA AGA GG-3’
	Reverse: 5’-GGC AGG ACA CCT GCA TAT TT-3’
